# Hereditary hemochromatosis beyond hyperferritinemia: Clinical and laboratory investigation of the patient’s profile submitted to phlebotomy in two reference centers in southern Brazil

**DOI:** 10.1590/1678-4685-GMB-2022-0230

**Published:** 2023-05-22

**Authors:** Nathalia Kersting, Juliana Cristine Fontana, Fabiane Pohlmann de Athayde, Fernanda Marcante Carlotto, Bruna Accorsi Machado, Cristiane da Silva Rodrigues de Araújo, Leo Sekine, Tor Gunnar Hugo Onsten, Sandra Leistner-Segal

**Affiliations:** 1 Universidade Federal do Rio Grande do Sul (UFRGS), Programa de Pós-Graduação em Medicina: Ciências Médicas, Porto Alegre, RS, Brazil.; 2 Hospital de Clínicas de Porto Alegre (HCPA), Serviço de Genética Médica, Porto Alegre, RS, Brazil.; 3 Universidade de Passo Fundo (UPF), Passo Fundo, RS, Brazil.; 4 Hospital São Vicente de Paulo, Serviço de Hemoterapia, Passo Fundo, Brazil.; 5 Hospital de Clínicas de Porto Alegre, Serviço de Hemoterapia, Porto Alegre, RS, Brazil.; 6 Universidade Federal do Rio Grande do Sul (UFRGS), Faculdade de Medicina (Famed), Departamento de Medicina Interna, Porto Alegre, RS, Brazil.

**Keywords:** Hyperferritinemia, diagnosis, Hereditary Hemochromatosis, *HFE* variants

## Abstract

Hereditary Hemochromatosis is a disorder characterized by iron deposition in several organs and hyperferritinemia. The most studied variants are linked to the *HFE* gene. In Brazil, surveys that characterize this population are scarce, with no sampling in the state of Rio Grande do Sul. Our objective is to carry out a data collection focusing on the profile of this population and the influence of the most frequently *HFE* variants. Two centers were enrolled: Hospital de Clínicas de Porto Alegre and Hospital São Vicente de Paulo. Patients with hyperferritinemia and undergoing phlebotomy were invited. Clinical data were collected, including *HFE* investigation. Among the descriptive data, the allele frequency of the C282Y variant (0.252) stands out, which differs from the national scenario. Systemic arterial hypertension was the most cited comorbidity. Differences between centers were observed, highlighting higher frequency of H63D cases in HSVP (p<0.01). Genotypes were stratified according to deleterious effect of C282Y variant. Higher transferrin saturation and number of phlebotomies were observed in the C282Y/C282Y cases (p<0.001). Positive family history for hyperferritinemia was more prevalent in compound heterozygotes (p<0.01). The results presented confirm the importance of encouraging such studies and reiterate the need for greater attention to this population.

## Introduction

Hyperferritinemia is a clinical condition verified by an abnormal increase of the serum ferritin parameter, which is not a usual test in routine examinations, but essential for the beginning of the investigation. The increased levels of this protein in the serum may be due to a variety of physiological situations or previously diagnosed comorbidities that may be directly or indirectly related to liver dysfunction. These include metabolic syndrome, chronic alcohol consumption, Gaucher disease, and reactive histiocytosis (Lukina et al., 1993;Fletcher, 1996;Regenboog et al., 2016;Sandnes et al., 2021). More commonly, in clinical practice, hyperferritinemia is found associated with acute and chronic viral liver infections, sepsis, cirrhosis, heart disease, and autoimmune diseases. The associated inflammatory response involves an increase in serum ferritin levels, which provides a defense against the growth of microorganisms by limiting the availability of circulating iron (Cherayil, 2011). Finally, another hypothesis for hyperferritinemia would be the production of ferritin in response to excess circulating iron as in Hereditary Hemochromatosis (HH) (Mateo-Gallego et al., 2010;Adams and Barton, 2011;Brissot et al., 2019).

HH is classified as an autosomal recessive disorder, with mostly European ancestry. This condition is characterized by increased intestinal iron absorption that can trigger excessive deposition in parenchymal cells, leading to cellular dysfunction and the clinical manifestations of the disease (Kane et al., 2021). It is classified into four main types depending on the underlying genetic mutation: human hemochromatosis protein (*HFE*) (type 1), hemojuvelin (*HJV*) (type 2A), hepcidin antimicrobial peptide (*HAMP)* (type 2B), transferrin receptor type 1 or 2 (*TFRC1/2*) (type 3), and ferroportin (*SLC40A1*) (type 4) (Adams, 2015;Wu et al., 2021). 

Thus, the hypothesis of a genetic disorder should be considered when a dysfunction in iron metabolism is observed. Since hyperferritinemia is considered a multifactorial clinical problem, epidemiological studies are essential to understand the needs of the population, both in terms of diagnosis, treatment and prevention of the associated consequences. However, the screening studies conducted to date at the national level, do not provide an adequate parameter of the proportion of people who are potential carriers of genetic variants related to iron metabolism. What is supported, both in terms of translational research and care, is the origin of these variants, which are most prevalent in Europe (Lucotte, 1998;Merryweather-Clarke et al., 2000;Distante et al., 2004). The most studied gene in HH is*HFE*, including the recurrent variants C282Y, H63D, S65C and its functional outcome. (Brissot et al., 2018). In routine care, only these three variants are analyzed, but testing is not available on the Brazilian Public Healthcare System, which is likely to have a direct impact on the accuracy of the clinical impression. The aim of this study is to conduct an epidemiological survey, describing the clinical and laboratorial characteristics, correlating these with the*HFE* gene variants.

## Material and Methods

### Population and procedures

*Study logistics and sampling*

A cross-sectional study was carried out from January 2019 to March 2020. Individuals with a confirmed diagnosis of hyperferritinemia were recruited from two care centers: the Hemotherapy Service of the Hospital de Clínicas de Porto Alegre (HCPA) and the Hospital São Vicente de Paulo - Passo Fundo (HSVP-PF). Contact with possible participants was made through the phlebotomies outpatient clinics; and recruitment took place as they were called to therapy sessions. The invitation was made by the research team on a scheduled day and time, without prejudice to assistance procedures. To be included in the study, participants had to be over 18 years of age and had been diagnosed with iron overload by laboratory examination at the time of initiation of the treatment. The parameter that includes this analysis is serum ferritin. Patients with multiple transfusions were not included, as hyperferritinemia is a consequence of the treatment.

*Database*

The construction of the database was carried out through an interview with the research participants and their prior authorization to consult information in electronic medical records. All participants signed the Informed Consent Form, with the right not to provide clinical data or not to know about the molecular result for the variants of the*HFE* gene. The collection of clinical variables respected the way they were described in the medical records, being categorized according to need within the statistical tests.

In addition to Ferritin at diagnosis, other parameters collected were Transferrin Saturation, age at treatment onset, Aspartate Aminotransferase (AST), Alanine Aminotransferase (ALT), body mass index, the presence of comorbidities, including alcohol abuse history, treatment history (phlebotomies or drug treatment) and the manifestation within the family of the same clinical condition. Regarding phlebotomies, in addition to their quantification up to the time of recruitment, a ratio was also calculated that accounts for the total number of the procedure by time, in months, of the patient under treatment.

*HFE genotypes*

Participants were asked to provide a molecular report with the investigation of C282Y, H63D and S65C variants in the *HFE* gene. In the absence of a previous result, a total blood collection was suggested, which was processed and analyzed at the Molecular Genetics Laboratory of the Medical Genetics Service-HCPA. At the end of the study, 214 genotypes were available.

*DNA extraction and genotyping*

DNA was extracted from 200 µL of whole blood according to a standardized protocol in a commercial Reliaprep Blood gDNA Miniprep System kit (PROMEGA©). The Real-Time PCR assay for allelic discrimination was performed using the TaqMan^®^ methodology (Thermo Scientific™) according to the manufacturer’s instructions. Specific inventoried and functionally tested probes for the C282Y, H63D and S65C variants were chosen. As positive and negative controls, patients already genotyped by external laboratories were used, and the results were replicated by the study methodology.

*Ethics statement*

All the samples are part of a project approved by our local Institutional Review Board (IRB0000921) from Hospital de Clínicas de Porto Alegre, which is recognized by the Office for Human Research Protections under the project number 2018-0542, and registered under the Certificate of Presentation for Ethical Appreciation (CAAE) #757318700005327. A written Informed Consent Form was obtained from all participants according to guidelines of the Good Clinical Practice.

### Statistical analysis

The collected data were submitted to Kolmogorov-Smirnov and Shapiro-Wilk normality tests. In view of the result, absolute and relative frequencies, means or medians were described, with standard deviation and 25th and 75th percentiles, respectively. Inferential analyses were conducted using parametric Student’s t, ANOVA’s tests; and nonparametric Kruskal-Wallis and Mann Whitney-U tests. Analysis of categorical variables followed the chi-square test. Regarding allele frequencies, a Poisson regression model with Robust Variance was used. P-values less than 0.05 were considered significant. As for multiple comparisons, post-test analyses such as Dunn’s test and residual analyses were designated. All tests were performed in SPPS Software version 18.0.

## Results

### Clinical data

**Figure 1 -:**
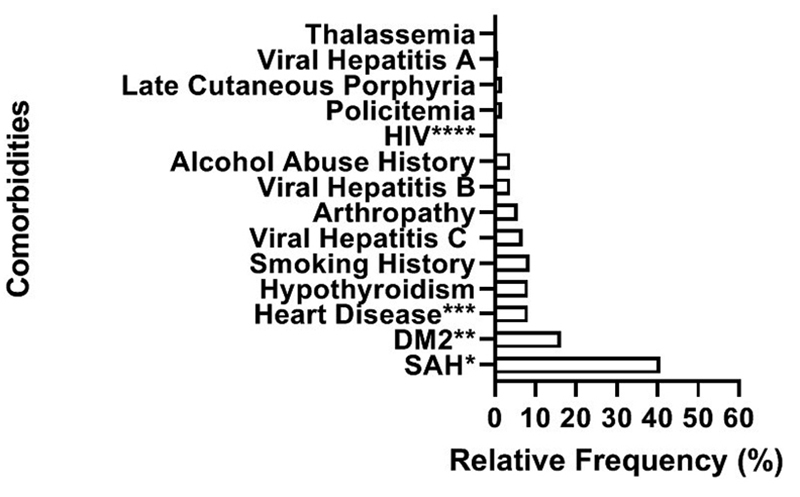
Graph showing the percentages of comorbidities reported by the 234 research participants enrolled in the study. *Systemic Arterial Hypertension, **Diabetes Mellitus (Type 2), ***Heart Disease (arrhythmia and cardiac insufficiency), ****Human Immunodeficiency Virus (two cases), Thalassemia (one case).

A total of 234 patients were recruited, 177 from the Hemotherapy Service - HCPA and 57 from the HSVP. The sample was mostly composed of men (79.5%) and the mean age at diagnosis was 54.1. With regard to family history related to HH, 35% reported at least one case to the research team or in their medical records. The median serum ferritin and transferrin saturation at diagnosis were, respectively, 1027.5 ng/mL and 52% (Table 1). Among the most cited comorbidities, systemic arterial hypertension, type 2 diabetes and heart disease were reported (Figure 1).

**Table 1 -:** Demographic and Clinical Characteristics of patients enrolled in the study at the HCPA and HSVP centers.

Variable	Sample (n= 234) median (p25;p75)¹
Sex (Male) ^*^	186 (79.5)
Positive Family History^*^	82 (35.0)
Age at diagnosis in years ^**^	54.1 (± 11.5)
Serum Ferritin at diagnosis (ng/ mL)	1027.5 (719.4; 1632.0)
Transferrin Saturation at diagnosis (%)	52.0 (39.4; 71.0)
Number of phlebotomies until recruitment	7 (3 ; 17)
Rate of phlebotomies per month	0.4 ( 0.2; 0.7)
BMI^2^	28.0 (25.9; 30.5)
AST^3^ (U/L)	27.0 (20.0; 39.5)
ALT^4^(U/L)	30.0 (21.0; 47.0)

^*^ absolute and relative frequencies (%); ^**^ mean (± standard deviation); ¹ 25th and 75th percentile; ² Body Mass Index; ³ AST= aspartate aminotransferase; ^4^ALT= alanine aminotransferase;

To ascertain any difference between the HCPA and HSVP samplings, the analyses followed with stratification for each center (Table 2). Among the variables with statistical significance, the earlier age of diagnosis in the HSVP center stands out, which may be related to other differences such as the rate of phlebotomy per month and other biochemical analyses regarding liver function.

**Table 2 -:** Clinical and laboratory data of research participants, stratified by study center.

Variable	HCPA Sample (n= 177) median (p25;p75)¹	HSVP Sample ( n= 57)	p-Value
Sex (Male) ^*^	134 (75.7)	52 (91.2)	**0.012**
Positive Family History^*^	59 (33.3)	23 (40.4)	0.334
Age at diagnosis in years ^**^	55.3 (±10.7)	50.6 (± 13.0)	**0.008**
Serum Ferritin at diagnosis (ng/ mL)	1068 (740.5; 1650.0)	977.0 (549.0; 1359.0)	0.057
Transferrin Saturation at diagnosis (%)	53.4 (39.0; 75.0)	50.0 (39.5; 65.0)	0.406
Number of phlebotomies until recruitment	8 (3; 20)	6 (0; 10)	**<0.001**
Rate of phlebotomies per month	0.4 (0.2; 0.7)	0.18 (0; 0.44)	**<0.001**
BMI^2^	27.7 (25.8; 31.0)	28.1 (26.2; 60.5)	0.834
AST^3^ (U/L)	27.0 (20.0; 42.5)	26.5 (19.2; 31.7)	0.161
ALT^4^(U/L)	32.0 (22.0; 54.5)	27.0 (21.0; 38.2)	**0.023**

^*^Absolute and relative frequencies (%), chi-square test analysis; ^**^Mean (± Standard Deviation), analysis by Student’s t test; ¹ 25th and 75th percentile, analysis by Mann Whitney-U test; ²Body Mass Index; ³AST= aspartate aminotransferase; ^4^ALT= alanine aminotransferase. p-value < 0.05 was considered significant.

Regarding the cited comorbidities, there were no differences between the centers. However, it is important to highlight that the HCPA center showed a greater tendency for the diagnosis of SAH (p=0.053). Further details are shown in Figure S1.

### Molecular analyses - Genotypes

**Figure 2 -:**
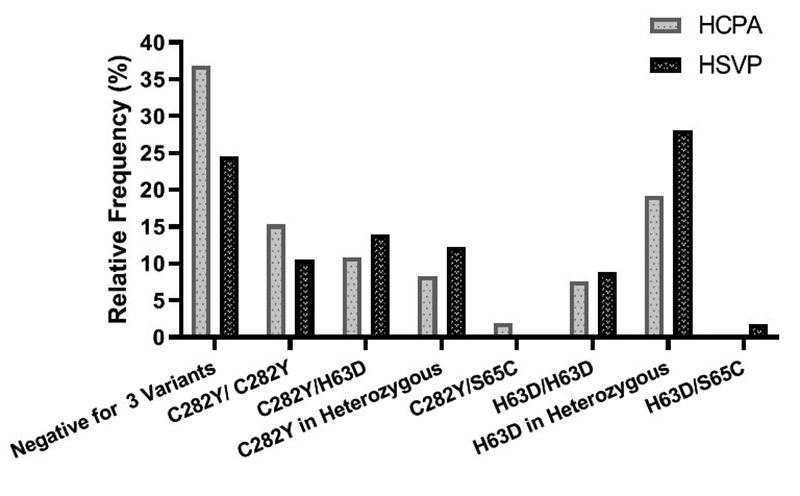
Description of the genotype frequencies of the sample under study, stratified by the research center listed. Statistical comparisons between centers were investigated by chi-square test. P-values 0.05 were considered significant. Genotype frequencies at each center in percentage (HCPA/HSVP): Negative for 3 variants (36.9/ 24.6); C282Y/C282Y (15.3/ 10.5); C282Y/H63D (10.8/ 14.0); C282Y/? (8.3/ 12.3); C282Y/S65C (1.9/ 0.0); H63D/H63D (7.6/ 8.8); H63D/? (19.2/ 28.1); H63D/S65C (0.0/ 1.8).

As already mentioned, not all participants agreed to proceed with the collection of whole blood for genotyping. Therefore, 214 samples were investigated, among which 33.6% were found to be negative for the three variants investigated in the *HFE* gene and 66.4% had at least one mutated allele. The possible genotypes and their frequencies are described as follows: H63D/? (21.5%), C282Y/C282Y (14%), C282Y/H63D (11.8%), C282Y/? (9.3%), H63D/H63D (7.9%), C282Y/S65C (1.4%), H63D/S65C (0.5%). With the stratification by research centers (Figure 2), at HCPA, the predominant genotype was negative for C282Y/H63D/S65C (36.9%), followed by H63D in heterozygosity (19.2%). At HSVP, heterozygous H63D was the most frequent (28.1%), followed by negative for C282Y/H63D/S65C (24.6%). Statistical differences in genotype frequencies were not found between the centers enrolled in the study (p=0.261).

*HFE variants*

**Figure 3 -:**
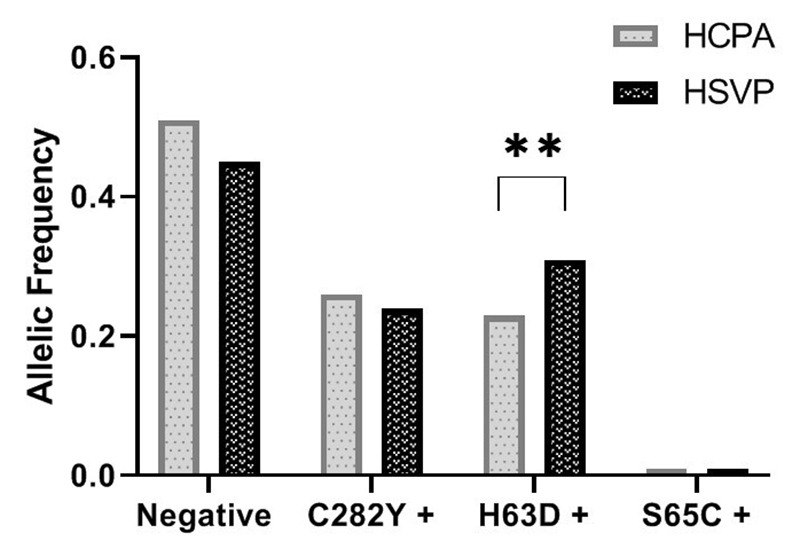
Description of allele frequencies for each *HFE* variant under investigation. Sampling was stratified by centers enrolled in the study. HCPA: C282Y (0.26), H63D (0.23) e S65C (0.01). HSVP: C282Y (0.24), H63D (0.31) e S65C (0.01). Statistical comparisons were performed using Poisson regression model with Robust Variance. P-values 0.05 were considered significant. **p <0.01.

Among the 214 research participants, the allele frequencies were as follows: negative for the 3 variants (0.492), C282Y (0.252), H63D (0.247) and S65C (0.009). When stratifying for the two research centers under study, a statistical difference was observed in H63D allele frequency (Figure 3).

Both in the scientific and healthcare environments, much is discussed about the variability in gene penetrance among the investigated variants, with C282Y being suggested as the one with the greatest causal effect, resulting in a greater loss of function of the encoded protein (Burke et al., 1998;Rossi et al., 2008;Rametta et al., 2020). Therefore, a stratification of possible genotypes was considered, in which C282Y in homozygosity and in compound heterozygosity with H63D and S65C were allocated in a group with possible differentiated clinical impact (group 1), followed by the other genotypes with at least one variant detected (group 2), and a third group which was negative for the variants investigated (group 3). The characteristics of each one are described in Table 3.

**Table 3 -:** Description of the variables under study, considering, in the stratification, the genotypes with the highest probability of clinical outcome.

Variable	Group 1 Sample (n= 58) median (p25;p75)^***^	Group 2 Sample (n= 84)	Group 3 Sample (n= 72)	p-Value
Sex (Male) ^*^	43( 74.1)	73( 86.9)	55( 76.4)	0,115
Positive Family History*	27 (46.6 )	33 (39.3)	19 (26.4)	0.051;
Age at diagnosis in years ^**^	51.3 (± 10.9)	54.6 (± 12.3)	54.6 (± 10.2)	0.180
Serum Ferritin at diagnosis (ng/ mL)	1082.0 ( 577.8; 1757.8 )	980.8( 714.6; 1350.0 )	1041.7 ( 734.5; 1504.3 )	0.329
Transferrin Saturation at diagnosis (%)	67.0 ( 50.7; 83.7)^a^	45.9 ( 35.5; 62.0)^b^	47.6 ( 34.6; 64.3)^b^	<0.001
Number of phlebotomies until recruitment	15.5 ( 6.0; 33.0)^a^	6.0 ( 3.0; 11.0 )^b^	6.0 ( 2.0; 13.3 )^b^	<0.001
Rate of phlebotomies per month	0.53 ( 0.25; 1.1 )	0.30 ( 0.15; 0.60 )	0.40 ( 0.19; 0.70 )	0.060
BMI^1^	27.4 (24.4; 30.4)	28.1 (26.0; 31.0 )	28.2 (26.6; 31.2 )	0.201
AST^2^ (U/L)	28.0 ( 20.7; 44,5 )	25.0 ( 20.0; 31,0 )	27.5 ( 20.0; 44.2 )	0.180
ALT^3^(U/L)	30.0 ( 21.8; 47.5 )	27.0 ( 21,0; 42,0 )	30.0 ( 22.5; 52.3 )	0.420

Group 1(C282Y/C282Y, C282Y/H63D, C282Y/S65C); Group 2(H63D/H63D, H63D/S65C, C282Y/?, H63D/?); Group 3 ( negative genotypes for the three variants); ^*^ absolute and relative frequencies (%), chi-square test analysis; ^**^ mean (± Standard Deviation), analysis by ANOVA test ; ^***^ 25th and 75th percentile, analysis by Kruskal- Wallis followed by Dunn’s multiple comparisons test, differences between groups are marked with the letters ^a^ and ^b^ underwritten; ¹ Body Mass Index, ²AST= Aspartate Aminotransferase; ³ALT= Alanine Aminotransferase. p-value < 0.05 was considered significant.

When the hypothesis test was performed for clinical variables, there was a significant difference between the genotype groups regarding transferrin saturation at diagnosis and number of phlebotomies until recruitment.

**Figure 4 -:**
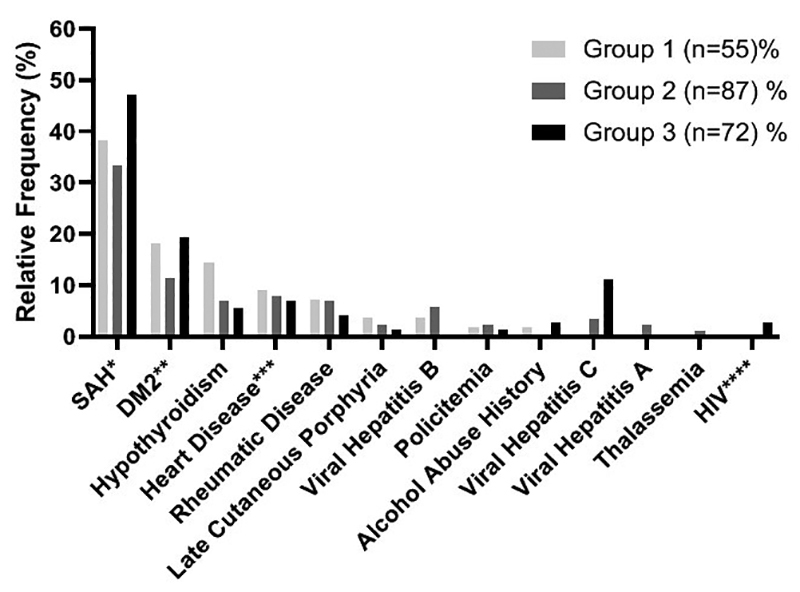
Relative frequencies of comorbidities for each genotypic stratification. *Systemic Arterial Hypertension, **Diabetes Mellitus (Type 2), ***Heart Disease (arrhythmia and cardiac insufficiency), ****Human Immunodeficiency Virus. Group 1(C282Y/C282Y, C282Y/H63D, C282Y/S65C); Group 2(H63D/H63D, H63D/S65C, C282Y/?, H63D/?); Group 3 ( negative genotypes for the three variants). Statistical differences between groups were investigated using the chi-square test followed by residual analysis. P-values <0.05 were considered significant. Adjusted standardized residual > 1.96 was used to confirm the difference between the groups in the HCV variable.

As for comorbidities reported by the research participants, a statistical significant difference was observed for the frequency of diagnosis of hepatitis C in group 3 when compared to groups 1 and 2 (p <0.05) (Figure 4).

**Figure 5 -:**
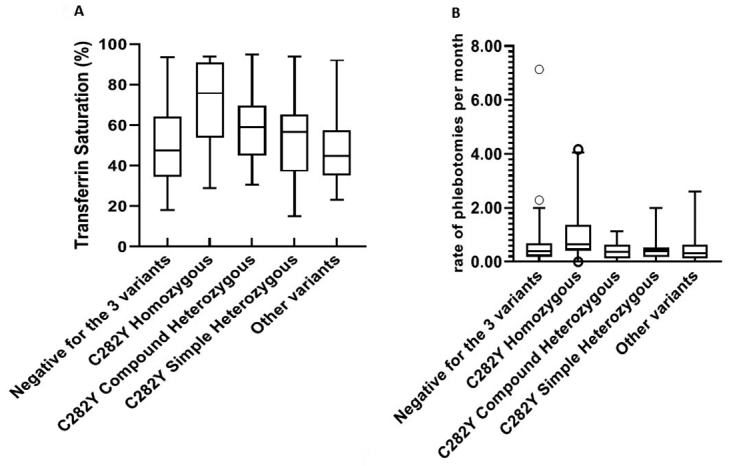
Description of Transferrin Saturation (%) and the rate of phlebotomies per month for each genotype, focusing on the C282Y variant. Statistical comparisons between groups were investigated by Kruskal- Wallis followed by Dunn’s multiple comparisons test. P-values ​​<0.05 were considered significant.

The *HFE* variants studied were submitted to similar inferential tests, with a new stratification. Different groups were considered with the presence of the main variant, substratifed for homozygous or heterozygous status, and the other genotypes were conglomerated into a single group. Since the study contains few cases with the S65C variant, the stratification considered only C282Y and H63D. In this hypothesis test, genotypes were tested only for positive family history for hyperferritinemia, transferrin saturation, rate of bleeds per month. The results described in Figure 5 show an increase of transferrin saturation and number of bleeds per month for C282Y in homozygous (Figure 5 A, B, p< 0.01). The positive family history, both in the analysis focusing on the C282Y variant, as in the case of H63D, showed a higher proportion of compound heterozygous, 60.7% and 69.2%, respectively (Table S1).

## Discussion

In the present study, the base criterion for eligibility of all participants was hyperferritinemia, a clinical condition that allows for a multitude of diagnoses. Therefore, for the evaluation of this sample, studies were taken into account that delimit the criteria for the diagnosis of HH (Pietrangelo, 2010;Brissot et al., 2011,^2018^;Powell et al., 2016).

The median results of ferritin (1027.5 ng/mL) and transferrin saturation (52%) presented are in line with what is considered a consensus in the literature for HH. The average age at onset of symptoms (54.1), the high percentage of men in the sample (79.5%) and the most cited comorbidities follow the same rationale (Table 1 and Figure 1). The concordance of these factors, plus the fact that 35% of the participants reported a family history, lead to the hypothesis that the regions covered by the centers included in the study would have a considerable prevalence of HH. This question is answered from the moment that at least 66.4% of the participants had a mutated allele for the investigated*HFE* variants, and that 35.6% had genotypes compatible with the genetic diagnosis (Figure 2). 

Comparisons with other studies reported in the literature are necessary. However it is important to point out that these studies, at the national level, are scarce. Besides, no other study in the same geographical region where our reserach was performed, has been reported. It is also worthy of note that, among the available studies, there is a disparity in the sample number and in the eligibility criteria (Bittencourt et al., 2002,^2009^;Oliveira et al., 2009;Santos et al., 2011;Leão et al., 2014). As shown inFigure 4, which compares different groups of genotypes, comorbidities such as alcoholism or other diseases that may affect iron metabolism do not exclude the diagnosis of HH. The same rationale should be made regarding laboratory data, since serum ferritin below 1000 ng/mL, or transferrin saturation <45%, do not exclude molecular diagnoses compatible with moderate hemochromatosis, which is the case of H63D genotypes in homozygosity or compound heterozygosity with S65C, allocated in group 2 ( Table 3).

Following a more general analysis, our sample was stratified for the two study centers included (Table 2). The results showed interesting differences from the clinical-assistance point of view, since the participants of the HSVP center have a lower age at diagnosis, which certainly reflects the other statistical differences that permeate the ALT test and affect the median number of bleeds per month. Given these differences, we can think of the inherent characteristics of each population: the ease of screening at the HSVP center may be related to the location in the countryside of the state, with a smaller contingent for care. Another difference to be pointed out falls into a more socio-economic context, since it is likely that the highest percentage of patients assisted via the Brazilian National Health Care System in the HCPA center have a more time-consuming process until reaching the diagnosis and treatment, due to the limitation of some resources. 

Some genotypes stood out in the sampling as being more prevalent. In both centers, heterozygous H63D had the highest percentage among participants with at least one mutated allele (Figure 3). The variant comprising this genotype, despite not being sufficient alone to conclude a molecular diagnosis, is an important phenotype modifier when added to exogenous factors (Aranda et al., 2010).The data presented inFigure 3 compare the research centers in terms of their frequency of variants, with a statistical difference being observed only for H63D, more prevalent in the HSVP center. Still on H63D, Pereira and colleagues reinforce its prevalence in Brazil, especially, in populations with strong European ancestry (Pereira*et al.*, 2001). The same study also delimits the allele frequencies of the C282Y variant, however, our sample differs with a greater number of patients with at least one copy of the mutated gene. The study reported by Santos and collaborators conducted an analysis of 51 patients with primary iron overload. Despite differences in eligibility criteria, genotypic and allelic frequencies were partially similar (Santos*et al.*, 2011). Regarding the S65C variant, the frequency of 0.01 was in line with previous result presented (Oliveira et al., 2009).

The clinical repercussions when only one copy of C282Y is present are not much discussed, since HH is an autosomal recessive disease. However, homozygous or compound heterozygous genotypes are often linked to clinical conditions that range from moderate to severe (Beutler, 1997;Olynyk et al., 2004;Whitlock et al., 2006;Allen et al., 2008). This variability in terms of clinical status is also seen in our study when we stratified the sample (Table 3). There is a difference in terms of transferrin saturation, which is essential for understanding the treatment in terms of prognosis and in the number of phlebotomies until recruitment. Taking these data into account, the importance of analyzing non-*HFE* genes is also highlighted, in which variants correlated with other HH isoforms, or phenotype modifiers, can explain the few differences between the groups.

Following the rationale used in other studies and the care routine, we focused on the C282Y and H63D variants, stratifying the groups for the different genotypic combinations, segregating the negative ones for that mutation and the negative ones for the three variants. The results remained similar to those observed for the genotypes allocated to group 1, more specifically, for C282Y homozygous cases with higher transferrin saturation values, as well as the number of bleeds per month (Figure 5). The analysis of these parameters corroborates with a previous study, which mentioned the greater risk of developing more serious signs and symptoms with this genotype and, consequently, a bloodletting schedule with a shorter interval (Evangelista et al., 2015). The high frequency of positive family history of hyperferritinemia reassures the importance of the C282Y variant; however, in this sample it was higher in the genotype in compound heterozygosity with H63D (Table S1). Biases about this result are possible, as they depend on the research participant’s memory. However, investigating this parameter as a research target in a disease with a possible genetic cause reinforces the fundamental idea that when treating a patient of this type, the impact can extend to the family, either in terms of understanding the clinical picture or in greater adherence to treatment.

## Conclusion

The pioneering of this study stands out, being unique in Rio Grande do Sul, the southern more state in Brazil. The greater need for investment in the diagnosis of these patients is also highlighted, since the need for molecular examination and continuous treatment of this population, which is prone to chronic comorbidities with a direct impact on quality of life, should be warranted.

## Supplementary material

The following online material is available for this article:

Table S1 - Tables comparing positive family history, stratifying the sample from the variants of interest C282Y and H63D.

Figure S1 - Graph showing the percentages of comorbidities reported by the 234 research participants enrolled in the study
