# Supplementary material for: Hereditary hemochromatosis beyond hyperferritinemia: Clinical and laboratory investigation of the patient’s profile submitted to phlebotomy in two reference centers in southern Brazil
Source: Genet Mol Biol. 2023 May 22;46(2):e20220230. doi: 10.1590/1678-4685-GMB-2022-0230 (PMC10206611; doi:10.1590/1678-4685-GMB-2022-0230)
Supplement: Table S1 - [file 1415-4757-GMB-46-2-e20220230-s1.pdf]

Genetics and Molecular Biology
https://doi.org/10.1590/1678-4685-GMB-2022-0230

Supplementary material to "Hereditary hemochromatosis beyond hyperferritinemia: clinical and laboratory investigation of the patient's profile submitted to phlebotomy in two reference centers in southern Brazil"

Table S1 – (1A & 1B) Tables comparing positive family history, stratifying the sample from the variants of interest C282Y and H63D. Statistical comparisons between groups using the chi-square test, followed by residual analysis. All tests consider a significant p-value <0.05. +Adjusted standardized residual > 1.96; - Adjusted standardized residual < -1,96.

1A

Variable	*C282Y/C282Y*	*C282Y/H63D OU S65C*<br>*N (%)**	*C282Y/___*	*Others variants*	*Negative*	*p-Value*
*Positive Family History**	*10 (33,3%)*	*17 (60,7%)+*	*9 (45%)*	*24 (37,5%)*	*19 (26,4)-*	***<0.001***

** absolute and relative frequencies (%);Statistical differences between groups were investigated using the chi-square test followed by residual analysis, +Adjusted standardized residual > 1.96; - Adjusted standardized residual < -1,96*

1B

Variable	*H63D/H63D*	*H63D/C282Y OR S65C*<br>*N (%)**	*H63D/____*	*Others variants*	*Negative*	*p-Value*
*Positive Family History**	*4 (23,5 %) -*	*18 (69,2%) +*	*19 (41,3%)*	*19 (35,8)*	*19 (26,4%)*	***<0.001***

** absolute and relative frequencies (%);Statistical differences between groups were investigated using the chi-square test followed by residual analysis, +Adjusted standardized residual > 1.96; - Adjusted standardized residual < -1,96*
