# Supplementary material for: Hereditary hemochromatosis beyond hyperferritinemia: Clinical and laboratory investigation of the patient’s profile submitted to phlebotomy in two reference centers in southern Brazil
Source: Genet Mol Biol. 2023 May 22;46(2):e20220230. doi: 10.1590/1678-4685-GMB-2022-0230 (PMC10206611; doi:10.1590/1678-4685-GMB-2022-0230)
Supplement: Figure S1 - [file 1415-4757-GMB-46-2-e20220230-s2.pdf]

Genetics and Molecular Biology
https://doi.org/10.1590/1678-4685-GMB-2022-0230

Supplementary material to "Hereditary hemochromatosis beyond hyperferritinemia: clinical and laboratory investigation of the patient's profile submitted to phlebotomy in two reference centers in southern Brazil"

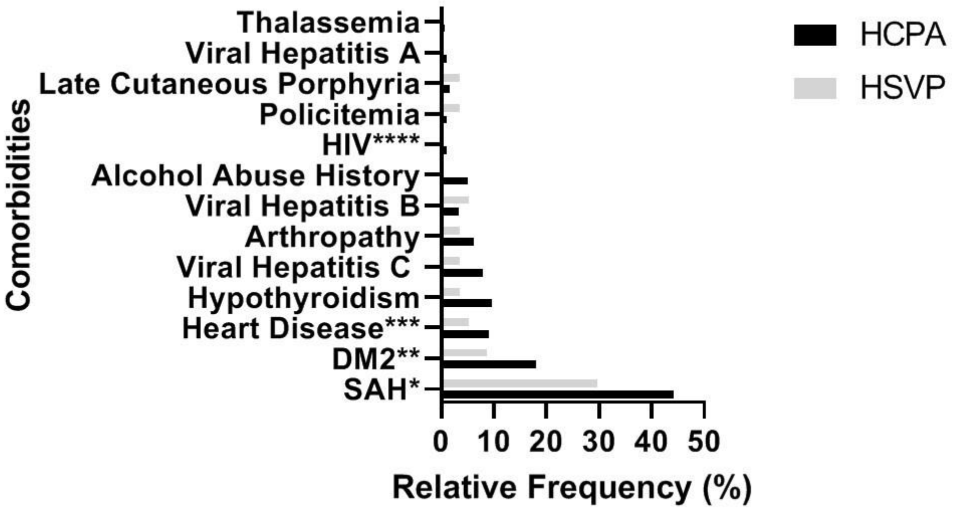

Figure S1 – Graph showing the percentages of comorbidities reported by the 234 research participants enrolled in the study. Sampling was stratified by centers enrolled in the study. *Systemic Arterial Hypertension, **Diabetes Mellitus (Type 2), ***Heart Disease (Arrhythmia and cardiac insufficiency), ****Human Immunodeficiency Virus
